# Composite Hydrogels for Bone Regeneration

**DOI:** 10.3390/ma9040267

**Published:** 2016-04-02

**Authors:** Gianluca Tozzi, Arianna De Mori, Antero Oliveira, Marta Roldo

**Affiliations:** 1 School of Engineering, University of Portsmouth, Anglesea Building, Anglesea Road, Portsmouth PO1 3DJ, UK; gianluca.tozzi@port.ac.uk; 2 School of Pharmacy and Biomedical Science, University of Portsmouth, St Michael’s Building, White Swan Road, Portsmouth PO1 2DT, UK; arianna.demori@port.ac.uk (A.D.M.); antero.oliveira@port.ac.uk (A.O.)

**Keywords:** biomaterials, regenerative medicine, hydrogels, bone regeneration, tissue engineering, mechanical properties, growth factors, statins, bisphosphonates, hydroxyapatite

## Abstract

Over the past few decades, bone related disorders have constantly increased. Among all pathological conditions, osteoporosis is one of the most common and often leads to bone fractures. This is a massive burden and it affects an estimated 3 million people only in the UK. Furthermore, as the population ages, numbers are due to increase. In this context, novel biomaterials for bone fracture regeneration are constantly under development. Typically, these materials aim at favoring optimal bone integration in the scaffold, up to complete bone regeneration; this approach to regenerative medicine is also known as tissue engineering (TE). Hydrogels are among the most promising biomaterials in TE applications: they are very flexible materials that allow a number of different properties to be targeted for different applications, through appropriate chemical modifications. The present review will focus on the strategies that have been developed for formulating hydrogels with ideal properties for bone regeneration applications. In particular, aspects related to the improvement of hydrogels’ mechanical competence, controlled delivery of drugs and growth factors are treated in detail. It is hoped that this review can provide an exhaustive compendium of the main aspects in hydrogel related research and, therefore, stimulate future biomaterial development and applications.

## 1. The Bone

The bone is a solid composite living material that represents the main constituent of the vertebral skeleton [1]. This dynamic tissue plays many important roles in the human body: first of all, by a joint action with the digestive and renal systems, it contributes to the regulation of the concentration of electrolytes, such as Ca and P [2,3]. Secondly, its hard and moderately elastic nature allows it to provide a framework for the support and attachment of softer tissues such as muscles, and for the protection of vital organs and the bone marrow. Finally, it provides all the necessary body support for locomotion and muscular contraction [4].

### 1.1. Bone Anatomy

The bone is typically composed of an outer layer of compact bone (also called cortical bone) embedding an inner spongy bone structure (also called trabecular or cancellous bone). Trabecular bone is formed by various trabeculae arranged in a honeycomb structure. The relative proportion between the cortical and trabecular bone varies with the skeletal segments and their function. The major difference between compact and trabecular bone lies in their porosity that ranges from 5% to 30% in the compact bone and from 30% to 90% in the trabecular bone. Long bones (*i.e.*, humerus and femur) are constituted by three regions: diaphysis, the central shaft; epiphysis, the bulbous extremities; and metaphysis, located between the two. In the middle of the shaft, a medullary (or marrow cavity) is present; this contains red bone marrow for hematopoiesis during infancy and additionally yellow marrow for energy storage during adulthood. Short bones include the tarsal and carpal bones; flat bones include the frontal and parietal bones of the cranium, ribs, scapula and pelvis; finally, irregular bones include the bones of the spine (vertebrae, sacrum and coccyx) and some bones of the skull such as sphenoid and ethmoid [5,6]. Bone shape is genetically determined in order to satisfy particular requirements according to the anatomic position and function. Furthermore, during life, bone shape is altered by the process known as remodelling, based on a combination of periosteal and endosteal apposition and resorption [7,8,9].

### 1.2. Chemical Composition

At a microscopic level, bone can be divided into three main components: matrix, cells and bioactive factors. The calcified bone matrix is formed by an organic protein-rich matrix (20% of dry weight), a mineral substance (65%) and water (~10%). The organic phase has a role in determining the form of the bone and affords resistance to tension. The major component of the organic matrix is collagen type I (90%); the remaining 10% is constituted of non-collagenous proteins, proteoglycans and phospholipids. Moreover, the matrix contains growth factors and enzymes such as phosphatase and metalloproteinase. Collagen fibrils are formed by three filamentous polypeptide chains in a helical configuration, which are stabilised through intra- and intermolecular cross-links that enhance the tensile strength of the fibrils. The non-collagenous proteins have different functions in the bone structure; phospholipids and proteoglycans have a regulatory effect in the calcification process [4]. The inorganic phase serves as an ion reservoir (predominately Ca, Mg, Na and P) and increases the strength of the bone due to the presence of apatite, carbonate, acid phosphate and brushite. The main inorganic component of the bone is hydroxyapatite [Ca_10_(PO_4_)_6_(OH)_2_] [10,11].

### 1.3. Histology of the Bone

The bone consists of cells from two cell lineages: mesenchymal stem cells (MSC) and hematopoietic stem cells (HSC).

1. MSCs are multipotent stromal cells that can differentiate into diverse cell types such as myocytes and adipocytes but also osteoblast progenitors, osteoblasts, bone-lining cells, chondrocytes and osteocytes. Osteoblasts are the most prevalent cell type in the bone and their function is to secrete matrix components, such as collagen I, in response to mechanical stimuli, and to promote the mineralisation of the bone matrix. Osteocytes, derived from osteoblasts, have mechanosensor and modulator (promotion of nerve growth) activities. Bone-lining cells are instead able to release enzymes to remove the layer of osteoids that covers mineralised matrix, allowing osteoclasts to attach and begin resorption.
2. HSCs are stem cells that give rise to blood cells such as monocyte, macrophages or platelets, but also preosteoclasts and osteoclasts. Osteoclasts are responsible for bone resorption; by the secretion of protons, they can lower the pH and so solubilize the mineral phase. These cells are responsible for an intricate balance between formation, maintenance and destruction of bone tissue. This equilibrium is maintained by mechanical factors and the action of cytokines and hormones, such as calcitonin and parathyroid hormone (PTH), which can control the levels of calcium and phosphate in the blood. Calcitonin is a thyroid hormone that reduces blood calcium levels by inhibiting osteoclasts and decreasing Ca resorption in the kidneys. Conversely, PTH is a hormone that increases blood calcium levels, acting upon the PTH1 receptor in bone and kidney, and the PTH2 receptor in the central nervous system, pancreas, testis, and placenta [6].

It is evident from the above brief description that the bone is an extremely complex tissue and that many factors can play a role in its physiology and function.

### 1.4. Bone Healing

**Figure 1:**
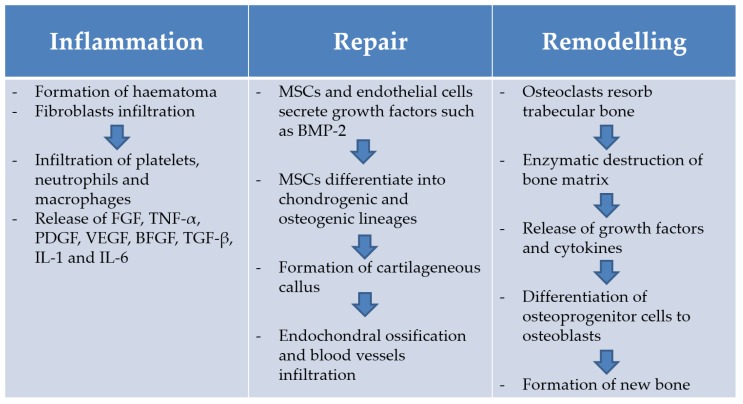
Bone healing process. BMP-2: bone morphogenetic protein-2.

The bone tissue possesses an intrinsic capacity of healing itself; the bone repairing process is an interplay of biomechanical, cellular and molecular factors [12]. If successful, bone regenerates itself with newly formed bone in children and in a mechanically stable lamellar structure in adults. These are formed after remodelling of newly formed woven bone [13]. The full understanding of this process may help researchers to develop new strategies to treat slowly healing or non-healing fractures [14]. First of all, when a fracture occurs, the following local tissue damage is observed: interruption of skeletal integrity, disruption of vascular structures and of nutrient flow at the fracture site, leading to a reduced oxygen tension and disruption of marrow architecture [15]. These are the prelude to the real bone regeneration process [16]. The bone healing process is divided into three phases: inflammation phase, reparative phase and remodelling phase (Figure 1) [13]. During the inflammation phase, blood vessel disruption causes the formation of a blood clot, called haematoma. Later, the immigrating platelets, neutrophils and macrophages release signalling molecules, such as fibroblast growth factors (FGF), tumour necrosis factor-α (TNF-α), platelet-derived growth factors (PDGF), vascular endothelial growth factor (VEGF), basic fibroblast growth factor (BFGF), transforming growth factor (TGF-β) and cytokines, such as IL-1 and IL-6 [15]. Then, IL-1 and IL-6 chemotactically attract mesenchymal cells precursors [16]. Finally, mesenchymal precursors proliferate and differentiate into the chondrogenic and osteogenic lineages [15]. During the second phase, or reparative phase, chondroblasts form hyaline cartilage while osteoblasts form woven bone that bridges and stabilises the bone wound. The so formed woven and hyaline cartilage is then substituted by lamellar tissue following a tissue-mineralisation process. The calcified tissue is then penetrated by blood vessels and the cartilaginous septum removed. The last phase is the remodelling phase during which the fracture callus is converted into new bone. In this phase, osteoclasts are responsible for resorption of trabecular bone, creating a shallow resorption pit, called Howship’s lacunae. Then, an enzymatic destruction of the bone matrix, promoted by the locally present cells, leads to the release of growth factors and cytokines. The latter induces the conversion of osteoprogenitor cells to osteoblasts that fill the resorption pits created by osteoclasts to create new bone matrix. Finally, the cells entrapped within the bone matrix generate osteocytes [15].

## 2. Bone Tissue Engineering

### 2.1. The Need for Effective Bone Repair Strategies: Economic, Social and Clinical Aspects

Over the past few decades, the prevalence of bone related disorders has steadily been on the rise [17]. The most notable cause for this is our increasingly aging population, however, other factors such as disease (*i.e.*, Paget’s disease or osteoporosis) and recurrent sports injuries are also relevant [18]. While these issues may have taken a back seat in comparison to the attention that heart disease and cancer have attracted, bone diseases are common and significant healthcare issues, and this has prompted a steady stream of research in the field of bone regenerative medicine [17].

Osteoporosis is one of the most common diseases leading to bone fractures; it affects an estimated 3 million people in the UK, and, as the population ages, numbers are due to increase [19]. The National Institute for Health and Care Excellence has published a guidance indicating that the direct cost from fragility related fractures in the year 2000 was estimated to be £1.8 billion, with the potential to increase to £2.2 billion by 2025 and to reach £6 billion by 2036, in the UK only [20].

Bone fractures also have a great impact on patients’ quality of life; for example, they can infringe on the ability of a person to live independently, when the fracture occurs along a load bearing bone, or they can affect self-esteem and social interaction when the spine and the posture of the patient are affected. They can also lead to high mortality in the elderly [19,20]. Moreover, co-morbidities can reduce the bone regeneration capacity with recurrence of delayed- or non-union fractures; these significantly prolong the recovery period and constitute a considerable societal burden [21].

### 2.2. Limitations of the Current Treatments

Current treatment protocols for complex bone fractures involve the use of auto-/allo-grafts or inert metallic/ceramic implants. Autograft implantation represents the golden standard for treatment of small bone defects [22]. This technique transplants the donor’s bone from a non-load-bearing site of the patient (*i.e.*, iliac crest) into the defect site in the same patient [23]. Bone autografting presents all the essential elements to heal a bone fracture, since the transplanted bone tissue has osteoconductive, osteoinductive and osteogenic properties. However, autografting shows some disadvantages such as high costs and limited available tissue from donor sites. Allograft is the second most common bone-grafting technique. This method involves the transplantation of bone from a different donor (*i.e.*, cadavers) and the main shortcomings are related to infections and demand of donor bone tissue [24]. When both auto- and allografts are not successful or adequate, different biomaterials are considered. Metallic devices such as plates/screws, rods and fixators are widely used, but despite their excellent mechanical properties, they are not bioactive or bioresorbable, thus limiting their performance and requiring additional surgical procedures in case of revision [25]. Moreover, due to stress shielding effects, they can cause bone resorption and consequent implant loosening [25]. Ceramics represent a valuable alternative due to their availability and adaptation to various applications. Calcium phosphate (CaP) cements, for instance, well resemble bone tissue chemical/functional properties, being both biocompatible and bioactive. However, low tensile strength and high brittleness are among the main drawbacks of such materials [26,27]. CaP showed ability in promoting bone repair, although they typically provide poor revascularisation/mineralisation, limited life-time, and inability to adapt to skeletal changes [28]. Furthermore, a single-phased material cannot efficiently guarantee bone growth, and tissue engineering approaches are needed [29].

### 2.3. Requirements for Successful Development of Bone Tissue Engineering Scaffolds

As reported by the pioneers Langer and Vacanti, the term tissue engineering (TE) defines “an interdisciplinary field that applies the principles of engineering and life sciences toward the development of biological substitutes that restore, maintain, or improve tissue or organ function” [30]. In general, TE aims at developing temporary 3D multicomponent scaffolds, also called composites, to induce the physiological regeneration of functional tissues (*i.e.*, bone), overcoming the downfalls of currently available biomaterials systems [25,31,32]. Scaffolds are firstly intended to work as fillers, occupying the available space in the damaged organ/tissue and slowly their programmed bioerosion/resorption activity allows them to provide a framework for growth of new tissue that finally (partially or completely) replaces the scaffold. Polymers’ bioerosion kinetics can be controlled, in order to be adjusted to *in vivo* bone formation rate, by modifying molecular weight, degree of modification, degree of crosslinking and by blending with different polymers. Saravanan *et al.* reviewed some conditions that can, for example, affect the rate of degradation of chitosan-based scaffolds [33].

An ideal scaffold for bone tissue engineering (BTE) must present the following three main properties [34]:

1. Osteoconduction refers to the growth of new tissue on the external and internal (pores) surfaces of the implant. This is greatly dependent on the physical form and chemical composition of the material. Factors such as hydrophilicity, porosity, biocompatibility and biodegradability of the material will affect its osteoconductive properties. For example, porosity (optimal pore size 200–350 µm) is crucial for allowing neovascularisation and diffusion of nutrients and gases required for the formation of the new bone [35]. Mechanical competence of the material is also important to provide an osteoconductive scaffold. This review will look at different approaches that have been studied in an attempt to improve the mechanical performance of hydrogel scaffolds.
2. Osteogenicity is the property of those scaffolds that contain osteoprogenitor cells and favour their adhesion and proliferation [36]. This review will look at the different types of stem cells that can be used in bone regeneration and strategies for their inclusion in hydrogel scaffolds.
3. Osteoinduction is the capacity of attracting immature cells to a healing site and stimulating these cells to develop into bone-forming cells. Materials that are osteoinductive are able to induce bone formation in ectopic sites [34]. This review will consider the complexity of controlled delivery of drugs and growth factors from bone regeneration scaffolds.

Osteointegration, defined at a histological level as the direct contact between the implant and the new bone without the formation of fibrous tissue and the ability to maintain a strong biomaterial/bone interaction over time, should be the overall outcome of the application of a scaffold that presents all the properties described above. Importantly, osteointegrated implants should re-establish the mechanical function of the repaired bone.

## 3. Hydrogels as Scaffolds and Delivery Platforms

In their letter to *Nature* in 1960, Wichterle and Lim investigated for the first time the role of hydrogels for biological use [37]; the following decades have seen a wealth of research aimed at exploiting the particular properties of hydrogels that allow their use for many purposes such as: matrices for tissue engineering and regenerative medicine, diagnostics, cellular immobilisation, separation of biomolecules or cells and barrier materials to regulate biological adhesion [10,38].

Hydrogels are three dimensional, hydrophilic, cross-linked polymeric networks [38,39]. They are able to swell without disintegrating and can absorb up to several times their dry weight in water [40]. Their high content in water makes them resemble the living tissue; therefore, they are ideal for a wide range of biomedical and pharmaceutical applications [41]. Hydrogels can be produced by different techniques: one-step methods like polymerisation and parallel cross-linking of multifunctional monomers, or multiple-step methods by the synthesis of polymer molecules characterised by the presence of reactive groups that originate cross-links in presence of low molecular weight crosslinking agents [42]. The crosslinking points are essential to avoid the dissolution of the polymer chains and the type and degree of crosslinking determine many of the properties of the hydrogel [41]; for example, crosslinking establishes the mesh size of the polymeric network and the water content that the gel can reach [42]. While the hydrophilic groups of the network chains attract and hold the fluid, the cross-links inside the structure impart an elastic force responsible for the solidity and stress-resistance of the hydrogel [41,42]. Hydrogel preparation techniques and considerations needed in their design have recently been reviewed by Sivashanmugam *et al.* [43].

Hydrogels are very flexible materials, amenable to different chemical modifications that can tune the desired properties according to the intended application. The present review will focus on the strategies that have been developed in the formulation of hydrogels with ideal properties for bone regeneration applications: osteoconduction, osteoinduction and osteogenicity.

### 3.1. Osteoconductive Composite Hydrogels: Strategies to Improve Hydrogels Mechanical Competence

It is widely accepted that scaffolds used for bone tissue engineering should be able to provide temporary mechanical integrity at the defect site immediately upon implantation. The mechanical properties of scaffolds should be tailored to match the demands of the implant site to decrease or avoid complications such as stress shielding, implant-related osteopenia, and subsequent re-fracture [44,45]. Consequently, the design of the scaffold will vary with the site of implantation and with the type of bone. However, specific mechanical requirements for scaffolds are still to be defined by the research community and clinicians.

The soft nature of hydrogels makes them unsuitable for applications where a certain mechanical competence is required and, although the mechanical properties of hydrogels can be increased by manipulating a number of parameters such as type and density of crosslinking [46], polymeric molecular weight, chemistry/concentration of the hydrogel precursors, it is not always possible to achieve the desired properties [47]. It must be also considered that some changes to the hydrogel formulation such as the use of high crosslinking density may lead to toxicity as well as a limit in the diffusion of loaded drugs, or the migration of cells and the exchange of gasses and nutrients that are essential for the healing of the fractured bone [48]. Therefore, designing hydrogel composites that are capable of synergising the biocompatibility and flexibility of the polymeric network and the structural support provided by the filler materials, can enhance the mechanical performance of hydrogels without affecting their beneficial properties. This results in a biomimetic approach where the physicochemical properties of nanostructured hybrid materials can stimulate cell growth and guide bone healing [49]. Different inorganic/organic composites for BTE have been studied; the most common fillers used are bioceramics (hydroxyapatite, tricalcium phosphate), bioglass particles, and carbon nanotubes. The reader is referred to the recent review by D’Este *et al.* for details of hydrogels specifically loaded with calcium phosphate [50]. The mechanical properties of nanocomposites can be controlled either by altering the properties of the matrix or of the nanofiller (*i.e.*, concentration and size) and interfacial bonding also has great importance as it may affect the effectiveness of load transfer.

#### 3.1.1. Hydroxyapatite

Biomedical applications of nano-hydroxyapatite (nHap) bioceramics have gained increasing interest due to their superior biological and biomechanical properties. Synthetic hydroxyapatite exhibits a strong affinity to host hard tissues due to the chemical similarity with mineralised human bone tissue [51]. Several studies have combined polymers and nHap to create composite hydrogels, merging the desirable properties of the different organic and inorganic phases in order to achieve a synergistic effect in the resultant composite properties including the enhancement of the mechanical properties. Li *et al.* developed nHap/polyacrylamide composite hydrogels that presented higher fracture tensile stress, higher extensibility, and higher compressive strength (35.8 MPa with 15% nHap *vs.* 22 MPa for pure gel) in comparison to the parent hydrogels; furthermore, these composites showed excellent shape recovery [52]. The authors justified the enhanced mechanical properties as the result of the chelating effect and the hydrogen bonding between the polymer chains and nHap particles. Natural polymers are also interesting biodegradable materials used in the formulation of scaffolds for bone tissue engineering, mainly due to their similarities with extracellular matrix, chemical versatility and overall good biological performance [53]. For example, silk fibroin displays slow biodegradation, adjustable mechanical properties, high permeability to oxygen and water vapour, resistance to enzymatic degradation, favourable processability and biocompatibility, and, as such, has attracted wide interest for a number of applications [54]. In addition, the mechanical integrity and low inflammatory response of silk fibroin ensure its role as a promising material for osteogenic applications. A nHap/silk fibroin composite hydrogel was developed by Ribeiro *et al.* [55]. In this composite, an increase in nHap concentration corresponded to an increase in mechanical properties of the composite with values of 90 and 100 kPa obtained for the compression modulus. Another natural polymer, agarose, extracted from marine red algae, can form thermoreversible gels via physical crosslinking. It is also a biocompatible and relatively bioinert material, which is promising for the formulation of gel matrices for biomedical applications [56]. Hu *et al.* studied nHAp/agarose composites and reported that with 70/30 organic/inorganic weight ratio the highest compressive strength value (~400 MPa) was obtained, while a 65/35 weight ratio afforded the highest elastic modulus (~1100 MPa, twofold that of pure agarose gel). This improvement in mechanical properties was justified by the formation of intermolecular hydrogen bonds between the two components, with a role also played by the average size (50 nm) of the well-dispersed spherical nHA particles [57]. Spherical porous hydroxyapatite granules prepared by freeze drying of nHA/polyvinyl alcohol/gelatin slurry droplets were encapsulated into oxidised alginate-gelatin-biphasic calcium phosphate hydrogel networks by Sarker *et al.* Also in this case, an increase of granules amount led to an increase in compressive strength due to mechanical interlocking of granules into the hydrogel matrix [58]. Chitin is another biocompatible, biodegradable and bio-resorbable biopolymer with also antibacterial and wound-healing abilities and low immunogenicity [59]. Chang *et al.* fabricated hybrid nHap/chitin hydrogels that exhibited porous structure, high mechanical strength and excellent biocompatibility [60]. Addition of nHap increased 10 fold (274 kPa) the reported compressive strength of chitin only hydrogel. The Young’s modulus of the hybrid hydrogel was ~320 kPa, compared to ~23 kPa of the chitin only hydrogel. Also, combinations of natural and synthetic polymers are abundant in the literature. For example, Arun Kumar *et al.* prepared a chitin/poly(caprolactone) hydrogel loaded with nHA. The gel maintained good injectability after addition of nHA, and presented a significant increase in elastic modulus [61].

#### 3.1.2. Bioactive Glass

Bioactive glasses or bioglasses are inorganic amorphous materials that present variations of composition based on the original bioglass (45S5): 45% silica (SiO_2_), 24.5% calcium oxide (CaO), 24.5% sodium oxide (Na_2_O), and 6% phosphorous pentoxide (P_2_O_5_) in weight percentage [62]. They display the ability to degrade at a controllable rate, releasing ions during this process and develop a carbonated phosphate surface layer that allows them to chemically bond to the native bone [63]. Bioactive glasses induce hydroxyapatite precipitation in the presence of a biological fluid, resulting in enhanced mineralisation of the bone tissue [63,64]. They also possess the ability to induce differentiation of mesenchymal cells into osteoblasts, to stimulate vascularisation and enhance osteoblast proliferation [65,66]. These properties are primarily dependent on the glass composition and microstructure [67]. Nevertheless, these materials are not suitable for load-bearing applications because of their intrinsic brittleness. Alone, they exhibit poor flexibility and fatigue strength, so their use in the formulation of composite hydrogels has been evaluated [68].

Gellan-gum represents a good candidate for the formulation of biocompatible scaffolds due to its non-cytotoxicity, biodegradability and hydrophilic nature [69]. However, similarly to other natural polymers, it presents relatively poor mechanical properties that narrow down its applications in bone tissue engineering. Although a combination of physical (*i.e.*, temperature) and chemical crosslinking approaches (*i.e.*, photocrosslinking) can produce gellan-gum hydrogels with tunable physical and mechanical properties without affecting their biocompatibility, the mechanical properties of the hydrogel alone are not suitable for bone tissue engineering [70]. Gantar *et al.* reinforced gellan-gum hydrogels with a nanoparticulate glass to improve the microstructure and the mechanical properties of the material [71]. The composite hydrogel scaffold containing 50% bioactive-glass exhibited a Young's modulus of ~1.2 MPa. Although this value is not sufficient to accommodate biomechanical loading, when compared with the gellan-gum alone, the incorporation of the bioglass significantly increased the Young's modulus from 0.4 to ~1.2 MPa and the failure stress increased from 0.02 to ~0.11 MPa. Photopolymerised poly(ethylene glycol) dimethacrylate hydrogels combined with bioactive glass particles were synthetised and characterised by Killion *et al.* who demonstrated that the incorporation of bioactive glass increases the mechanical strength of the composite material, with a synergic action in which the bioactive glass absorbed the initial compressive load and the polymeric matrix distributed the load between the reinforcement [72]. Lacroix *et al.* reported the synthesis of a bioactive glass/gelatin composite scaffold with well-controlled porosity [73]. Compressive loading was performed on pure bioactive glass and composite scaffolds: the glass foams presented a step-by-step cracking characteristic of brittle materials, while the deformation of the gelatin-bioactive glass composite remained in the linear elastic regime. Besides these improved mechanical properties of the composite, its *in vitro* bioactivity was found to be as high as pure bioactive glasses.

#### 3.1.3. Carbon Nanotubes (CNTs) and Other Carbon Materials

CNTs are allotropes of carbon composed of rolled up graphene sheets. According to the number of sheets concentrically aligned to form one-dimensional nanostructures, single-wall nanotubes (SWNTs) or multi-wall nanotubes (MWNTs) can be obtained [74]. CNTs present interesting and unique properties such as extreme toughness, high electrical conductivity and high surface area [75]; and are of interest in tissue engineering as they can support the building of flexible and porous structures similar to the extracellular matrix (ECM), an environment in which cells physiologically migrate and proliferate to form tissues and organs [76]. Seo *et al.* developed a degradable membrane composed by chitosan/silica incorporating functionalised-carbon nanotubes (f-CNT) for guided bone regeneration [77]. In their study, the incorporation of 2% f-CNT substantially enhanced the mechanical properties (tensile strength and elastic modulus) of CNT/chitosan/silica membranes in comparison with chitosan/silica or bare chitosan membranes, while it did not have an effect on elongation rates. In our lab, thermosensitive chitosan gels were reinforced with chitosan grafted CNTs. It was found that CNTs not only increased the resistance of the gels to compression but, due to their thermal properties, they also had a major role in determining the time of gelation of the composite gels reducing it from around 1 h for chitosan only gels to about 7–8 min [78]. Nanocomposites of the biodegradable and biocompatible polymer poly(propylene fumarate) (PPF) reinforced with three carbon nanostructures (SWNTs, ultra-short SWNTs (US-tubes) and fullerenes (C60)) were fabricated by Sitharaman *et al.* The nanostructure size and surface area effects on the rheological properties of un-cross-linked PPF dispersions, as well as the mechanical properties of cross-linked nanocomposites were investigated as a function of the nanostructure concentration [79]. The US-tube nanocomposites showed the best mechanical enhancement effects. The mechanical properties for US-tube nanocomposites peaked at concentrations of 0.5% and significantly enhanced flexural and compressive mechanical properties (up to 200%), when compared to the pure PPF. The study concluded that US-tubes and SWNTs contributed to better mechanical reinforcement than C60, due to the higher aspect ratios and larger surface areas suggesting that the surface area of carbon nanostructures may be more important than size for the mechanical reinforcement. Furthermore, it was suggested that the fibril-like morphology of the SWNTs and US-tubes might also contribute to the improved mechanical properties of the nanocomposites.

Other carbon structures have also been evaluated; Lalwani *et al.* compared the efficacy of two dimensional (2D) carbon and inorganic nanostructures as reinforcing agents of crosslinked PPF composites: single-walled graphene oxide nanoribbons, multi-walled graphene oxide nanoribbons, graphene oxide nanoplatelets, and molybdenum di-sulfite nanoplatelets (MSNPs) [80]. The mechanical properties (compressive modulus, compressive yield stress, flexural modulus and flexural yield stress) of all the 2D nanostructure-reinforced nanocomposites as a function of 2D nanostructure concentration (between 0.01% and 0.2%) were significantly higher in comparison to PPF. It was found that the mechanical reinforcement is closely dependent on the nanostructure morphology and follows the order nanoplatelets > nanoribbons > nanotubes. The inorganic 2D nanostructure MSNPs showed better mechanical reinforcement than 1D or other 2D carbon nanostructures. The 2D nanostructures increased the crosslinking density of PPF nanocomposites compared to 1D nanostructures. Accordingly, the authors suggested that harnessing the reinforcing potential of 2D nanostructures could lead to an entire new class of ultra-strong, lightweight biomaterials for tissue engineering applications [80].

The inorganic components considered above enhance the mechanical properties leading to reinforcement of the scaffold structure. Although the composite strategy is very promising, so far the scaffolds obtained present mechanical properties far from resembling human bone. Limited literature links properties such as fracture toughness or energy to failure of the composites. There are studies that provide some mechanical analysis of the composites produced; however, few provide all the characteristics that aid interpretation and comparison of the data. Data for bending/tension and for measures of fracture toughness are not common, consequently creating several difficulties in producing comparisons across studies as shown in Table 1 [81]. It is necessary to develop standard procedures regarding the mechanical analysis of these materials, in order to facilitate comparison between different studies and obtain a complete characterisation of the materials. One of the biggest remaining challenges for composites it is to define and achieve appropriate mechanical requirements of scaffolds for load-bearing defects.

### 3.2. Osteoinductive Composite Hydrogels: Controlled Delivery of Drugs and Growth Factors

While a well-designed scaffold can favour new bone formation in healthy patients by simply providing a support for the growth of new tissue, the compromised bone homeostasis in osteoporosis and other bone conditions hinders the healing process and requires the use of drugs and growth factors to stimulate bone regeneration activity. In many cases, controlled and sustained release of these pharmaceutical entities is required and hydrogels present an ideal platform for their release. Several classes of drugs and different growth factors have been evaluated; these are discussed in detail below.

#### 3.2.1. Bisphosphonates

Bisphosphonates (BPs) are one of the most important drug classes for treating bone tissue diseases such as metastatic bone disease and osteoporosis [83]. They are carbon-substituted pyrophosphate analogues [84] and they can reduce bone resorption, causing loss of osteoclastic activity and accelerating osteoclast apoptosis by inhibiting farnesyl pyrophosphate synthase (an enzyme in the 3-hydroxy-2-methylglutaryl-coenzyme A (HMG-CoA) reductase pathway) [85]. The bisphosphonates currently used in clinical practice are all biologically stable due to presence of a carbon atom connecting the two phosphates (P-C-P) [86]: alendronate, clodronate, ibandronate, risedronate and zoledronate [87,88]. Each bisphosphonate presents a certain bone binding affinity: the higher the affinity, the stronger the binding and slower the release and *vice versa* [85]. These molecules exhibit high affinity for the mineralised bone matrix where they can be retained for several years, leading to potent pharmacological effects on the bone [89]. The order of efficacy in inhibiting farnesyl pyrophosphate synthase is zoledronate > risedronate > ibandronate > alendronate. The order in the kinetic binding affinity to hydroxyapatite (Hap) is clodronate < etidronate < risedronate < ibandronate < alendronate < pamidronate < zoledronate [90]. Controlled trials have highlighted that BPs reduce the incidence of vertebral fractures by 40%–50% and non-vertebral fractures by 20%–40% [91]. Unfortunately, these drugs present some drawbacks when orally administered, in particular low adsorption (0.6%–1.5%) and high toxicity (esophageal disease, atrial arrhythmias, osteonecrosis of the jaw and atypical femur fractures) [92].

Hydrogel systems have therefore been developed to allow controlled local delivery with minimal invasiveness and time-persistence at the targeted area of the affected tissue. Despite the promising effects of these drugs, BPs’ local release is still poorly explored. A common strategy investigated for BPs controlled local delivery is to combine the controlled release from a biodegradable particulate system with the diffusion barrier provided by a hydrogel scaffold. For example, Posadowska *et al.* encapsulated alendronate in PLGA (poly(lactide-*co*-glycolide)) nanoparticles by a solid/oil/water emulsification method (final drug loading 5%) [93]. The obtained nanoparticles (average diameter 230 nm) were then suspended in a gellan-gum (GG) hydrogel and the hydrogel matrix was cross-linked with calcium ions to improve stiffness. PLGA was chosen because it is characterised by a tunable degradation rate and FDA-approved, while GG is a low cost anionic natural polysaccharide widely used for pharmaceutical purposes. This formulation allowed obtaining a release of 17% of the drug after one day, while the entire encapsulated drug was released within 25 days. Moreover, *in vitro* studies confirmed cytocompatibility of the formulation with MG-63 osteoblast-like cells and its capacity to inhibit osteoclastic differentiation.

Furthermore, the high affinity of BPs for HAp can be exploited to facilitate controlled release of these drugs from hydrogel scaffolds. Verron *et al.* [89] formulated a suspension of zoledronate (Zol) granules loaded into calcium deficient apatite (CDA-Zol) in a cellulosic-derived hydrogel. CDA was chosen to reinforce the osteoporotic site, while Zol to inhibit osteoclast resorption activity. The implantation of CDA-Zol in distal femurs of osteoporotic female rats showed a significant increase in bone volume fraction (BT/TV) and improved trabecular architecture compared to apatite alone. Further histological examination did not show the presence of abnormal tissue (*i.e.*, fibrosis, necrosis, and granuloma) in the newly-mineralised area.

#### 3.2.2. Statins

Statins are competitive inhibitors of HMG-CoA reductase. They are generally used for lowering serum cholesterol [94], blocking the conversion of HMG-CoA to mevalonate. Hence, they are commonly used for treating diseases such as hyperlipidemia and arteriosclerosis. More recently, some studies have highlighted statins ability to increase new bone formation in cell cultures and in animal models [95,96,97]. This anabolic effect has been firstly elucidated by an increased mineral density in bones of type 2 diabetes patients when treated with statins [98]. Bradley *et al.* demonstrated that these effects are associated with an increased expression of bone morphogenetic protein-2 (BMP-2) and nitric oxide synthase [99]. However, clinical use of statins is limited by the really low systemic availability (~2%) and serious side effects such as liver toxicity, acute hepatic failure and episodes of myalgia [100,101,102,103]. Hence, local delivery (bypassing hepatic metabolism) could lead to higher concentrations at the bone and a reduction of the side effects [101]. In this context, the formulation of an appropriate scaffold is really important to achieve appropriate release kinetics and a local action. Simvastatin (SIM) is recognised as the most potent statin in stimulating bone growth, with a biological half-life of 1–3 h and susceptibility to cytochrome P450 metabolic activity [101]. This fungal metabolite induces osteoblastic differentiation by increasing the expression levels of osteogenic markers such as alkaline phosphatase, osteocalcin and osteopontin [104]. Many studies have been carried out for testing different materials and developing a suitable hydrogel scaffold [105].

Tanigo *et al.* successfully obtained a controlled release of SIM from gelatin/micelles composite hydrogels [106]. In particular, SIM was firstly water-solubilised into lactic acid oligomer grafted gelatin micelles. These micelles were then loaded into gelatin hydrogels, with tunable degradation rate based on degree of crosslinking. The system allowed controlled release of SIM over 20 days and enhanced the effect of the drug on bone regeneration in a rabbit tooth defect model. Sukul *et al.* prepared a three components hydrogel scaffold for the controlled release of SIM composed of: β tricalcium phosphate (β-TCP), an osteoconductive material; nanofibrillar cellulose, a slow degrading polymer that can improve control over the release rate of the gel; and crosslinked gelatin as the main hydrogel matrix [107]. The presence of nanofibers and β-TCP produced a scaffold with osteoconductive properties and able to better control the drug release over a period of time exceeding 30 days. Furthermore, a concentration of 0.5 µM SIM was identified as the most effective, both *in vitro* (highest proliferation and differentiation of rat MSCs) and *in vivo*, in a 8-mm rat calvarial defect with a 33% new bone formation after 8 weeks treatment (compared to 25% and 17% obtained with lower and higher concentrations, respectively). Another well studied statin for bone regeneration applications is fluvastatin. Benoit *et al.* explored the covalent binding of this drug to the gel forming polymer, as a strategy for controlled release [100]. They used a biodegradable poly(lactic acid) spacer to load fluvastatin to a poly(ethylene glycol) dimethacrylate hydrogel. The release rate of the drug was controlled by the length of the spacer; the longer the spacer, the quicker the release due to the presence of a higher number of hydrolysable bonds and an increased probability of release. This system showed potential for controlled release over 40 days, *in vitro* the drug was able to induce hMSCs differentiation, increase BMP-2 production and facilitate calcium deposition.

#### 3.2.3. Growth Factors

Growth factors such as Bone Morphogenic Protein (BMP) are clinically relevant therapeutics for tissue regeneration in musculoskeletal conditions. However, their current use has shown very limited success because, even though collagen scaffolds have been developed for the controlled release of BMP, the protein short half-life and diffusion to other tissues requires the use of very high doses (10 µg) rendering the treatment costly and unsafe. Complications such as ectopic bone formation, compromised airways function, swelling at the surgery site and neurological side effects have all been reported in spinal fusion application of BMP [108,109]. Thus, developing new strategies for the formulation and delivery of these growth factors could address the unmet clinical need for safe use of bone inducing biomaterials. Furthermore, research has demonstrated that physiological bone healing involves several different factors released at different times (Figure 1), indicating that release of a single growth factor would be an oversimplification in an attempt to mimic the physiological response [36].

As seen in the case of controlled release of smaller drugs, also for the delivery of growth factors, composite gels are used. The strategies for sustained release are very similar; in general, a biodegradable or bioerodible multiparticulate system is introduced in the gel to further delay the release of the entrapped macromolecule. Strategies such as core-shell and layer-by-layer deposition are commonly used for the encapsulation of one or more growth factors [36].

The limitations of the current clinically available BMP-2 formulations have induced a lot of research into improving the mechanism of controlled release of this protein, to provide better release and address its short half-life and rapid local clearance. A commonly used strategy is that of exploiting the known affinity of BMP-2 for heparin, therefore, including heparin into hydrogels. A study conducted by Bhakta *et al.* demonstrated that both the initial burst, from the plain hyaluronic acid gel, and the prolonged sustained release of BMP-2, obtained with the addition of heparin to hyaluronic acid gels, were essential for efficient bone formation, and suggested that a compromise between diffusion and affinity release must be found for best performance of the gel [110]. Chung *et al.* exploited the same strategy in their fibrin gel loaded with heparin-functionalised nanoparticles in which the presence of heparin was instrumental in reducing the rate of release of BMP-2 and enhance quantity and mineralisation of the newly formed bone *in vivo* [111]. Recent advances have looked at introducing multiple control systems. This is, for example, the case of dual interaction nanoparticles developed by Seo *et al.* [112]. They proposed a hydrogel that forms *in situ* after injection of polymeric nanoparticles that bind to BMP-2 by both ionic and hydrophobic interactions; and demonstrated that dual interactions were essential in generating a more effective formulation *in vivo*.

As recently reviewed by Bayer *et al.*, growth factor delivery is moving towards the sequential release of two factors from individual scaffolds with the release of a factor involved in angiogenesis in the first release phase, followed by a growth factors such as BMP-2 in a second release phase [36]. Table 2 summarises the growth factors involved in bone healing that have been most commonly studied for bone tissue engineering applications.

Dyondi *et al.* carried out a study to investigate the effect of dual growth factor release [113]. They developed a gellan-xanthan gum gel loaded with chitosan nanoparticles. BMP-2 was adsorbed on the surface of the nanoparticles while FGF was loaded in the gel. A comparison between single and dual growth factor release was carried out *in vivo* and showed that the combined release of the two growth factors resulted in a higher ability to promote osteoblast proliferation and differentiation. Interestingly, the gels also presented antimicrobial properties.

Doubtlessly, as demonstrated by the examples reported above, due to their physical and chemical flexibility, hydrogel formulations, and, even more so, hydrogel composite formulations, represent an essential tool in the development of controlled release systems for the local delivery of therapeutic molecules to the bone.

### 3.3. Osteogenic Composite Hydrogels

Osteogenesis is characterised by a high number of cells compared to the mature bone tissue. These are osteoprogenitor cells that produce the extracellular matrix, which will support bone formation and will be later resorbed [114]. A good strategy to be employed in bone regeneration is that of recreating the environment in which osteogenesis takes place; this can be mimicked with a 3D hydrogel network that allows osteoprogenitor cells to proliferate and differentiate into osteoblasts. In fact, hydrogels are able to create a complex and dynamic network that replicates the characteristics of the extracellular matrix providing physical structure, mechanical integrity and biocompatibility with the host tissue [115,116]. Studies have investigated the use of different cell types that will be considered in detail below [12].

#### 3.3.1. Mesenchymal Stem Cells

Stem cells are undifferentiated cells capable of self-renewal and differentiation into specialised cells [102]. Mesenchymal stem cells (MSCs), otherwise known as bone marrow stromal cells, are multipotent adult stem cells which can be found in the bone marrow, among other sites [41,98]. MSCs can proliferate *in vitro* and differentiate into diverse mesenchymal lineages (adipocytes, chondrocytes, myocytes, osteoblasts and tenocytes) in response to appropriate signaling by chemicals, growth factors and hormones [98,117]. Interestingly, MSCs possess trophic factors that suppress the local immune system, decreasing the risk of autoimmune rejection and promoting local vascularisation [12,118,119]. Moreover, they are easy to isolate from bone marrow and to manipulate. In fact, they can be enriched to obtain a relatively pure population of cells and differentiate into osteoblasts that are capable of secreting extracellular matrix [102]. Many researches are focusing on the identification of a suitable three-dimensional scaffold for cell transplantation to promote the localised healing of the desired tissue. The aim is to develop a scaffold that is both biodegradable and injectable, in order to allow the growth of the new tissue as degradation of the polymer scaffold occurs, and to perform a single minimally invasive procedure [120]. However, the shortcomings of current methods underline the need for combining our understanding of what cell types can form bone and what scaffold best facilitates the differentiation of these cells [121]. Several hydrogels including alginate, collagen, fibrin glue, hyaluronic acid, oligo[poly(ethylene glycol) fumarate], pluronic F127 and silk fibroin have been studied to encapsulate MSCs and to promote their osteogenic differentiation [102,104,122].

Nuttleman *et al.* successfully proposed the encapsulation of hMSCs into a photocrosslinkable, injectable scaffolding system based on a dimethacrylated PEG (MW 4.6 kDa) as the hydrogel precursor [123]. These gels presented an excellent cytocompatibility (relative cell survival: 99%) and osteogenic-specific differentiation was confirmed by the expression of osteonectin, osteopontin and alkaline phosphatase genes. Finally, a staining procedure revealed extensive mineralisation of the PEG hydrogels. Dimethacrylated PEG is also suitable to prepare scaffolds via the novel approach of bioprinting. Gao *et al.* [124] loaded hMSCs on photocrosslinked PEG-hydrogel scaffolds enriched with natural cell binding motifs (Arg-Gly-Asp or RGD peptide and matrix metalloproteinase-sensitive peptide). The bioprinting process allowed them to condense multiple steps of scaffold fabrication into one single step and to obtain a matrix that leads to a homogeneous development of bone and cartilage. Moreover, the bioprinted PEG-peptide scaffold showed excellent mineral and cartilage matrix deposition and the addition of the peptides showed a sustained effect over time on cell differentiation compared to the polymer only gel.

Another extensively studied polymer for BTE is hyaluronic acid (HA), a naturally derived, linear, high molecular weight polymer and one of the major components of ECM [125]. Many studies have been carried out in order to modify its structure, allowing the attachment of therapeutic drugs, functional groups, crosslinkers, and other bioactive moieties to HA [118,119]. Kim *et al.*, following a similar approach to the one by Nuttleman *et al.* described above, prepared an acrylated HA hydrogel for loading BMP-2 and hMSCs [125]. The formulation had a gelation time of 10 min at physiological conditions; however, mechanical testing (complex modulus 1.8 kPa, elastic modulus 1.8 kPa) revealed that this hydrogel was not strong enough for load bearing applications. Even though the formulation presented a relatively low *in vitro* cell viability (72% within two days, increased to 81% with the addition of BMP-2), the *in vivo* results showed that it was able to induce angiogenesis and osteogenesis. The HA hydrogel alone demonstrated good compatibility but no activity, while the addition of BMP-2 and hMSCs revealed a synergistic effect with formation of thicker and denser new bone, compared to the hydrogels containing only one of the two components.

Collage type I matrices are currently used in clinical practice for example for the delivery of BMP-2 (InductOs); collagen is one of the most abundant fibrous proteins in the human body, found in tendons, ligaments, bone, teeth, skin, arteries and, in general, in extracellular matrix [126]. Collagen is a common tissue culture matrix due to its ability to facilitate cell attachment and its cell-based degradation. The ability of collagen type I hydrogels to favour attachment, migration and proliferation of rat bone marrow stromal cells was demonstrated by Hesse *et al.* [127].

Fibrin glue is another polymeric gel already exploited in the clinic, which has attracted interest in bone regeneration applications for the implantation of stem cells [128]. Seebach *et al.* implanted fibrin glue hydrogels loaded with rat derived MSCs into rat femoral bone defects to test host cell-recruitment, immunomodulation and tissue regeneration [116]. Results highlighted fibrin-MSC composite promoted host macrophage invasion, in comparison to cell-free fibrin hydrogels, due to the MSCs’ expression of trophic factors (e.g., IL-6, VEGF and MIP-2). Then, MSCs seemed to stimulate femoral bone healing, despite a light induction of a pro-inflammation process (TNF-α and IL-1β).

MSCs’ harvesting from the bone marrow requires painful procedures of aspiration from the iliac crest or from bone marrow biopsies that can anyway give low cells yields [121]. Hence, due to these practical constraints, researches have focused on the use of multi-lineage mesenchymal progenitor cells from other sources.

#### 3.3.2. Adipose Derived Stem Cells

Human adipose-derived stem cells (ASCs) are a subset of MSCs that can be found in human adipose tissue [129]. The adipose tissue is a highly complex tissue that consists of mature adipocytes (90% of the total volume) and a stromal vascular part composed by pre-adipocytes, fibroblasts, vascular smooth muscle cells, endothelial cells, resident monocytes/macrophages lymphocytes and ASCs [130]. ASCs are an interesting cell-lineage for regenerative medicine since they present morphological, immunological and phenotypical properties similar to stem cells isolated from bone marrow and umbilical cord blood, with some advantages [129,131,132]. In fact, ASCs have the potential to differentiate into bone, cartilage, tendons, skeletal muscle and fat under specific conditions and they are easy to access, abundant in the subcutaneous adipose tissue and isolated by an uncomplicated enzyme-based procedure [132]. The combination of ASCs with biomaterial scaffolds is currently a very promising strategy for restoring tissue functions, but further advances are needed.

Heo *et al.* [133] loaded ASCs on a photo-curable gelatin-gold nanoparticle (GNPs) hydrogel in order to study the effects of GNPs on stem cells. According to recent studies, GNPs have been shown to have a positive effect on osteogenesis of MSCs and MC3T3-E1 osteoblast-like cells, other than presenting low costs and non-toxicity [134,135]. In Heo’s study, the hybrid hydrogel was obtained by irradiation of the mixture of the photo-initiator and methacrylated gelatin leading to successful loading of the GNPs inside the hydrogel network. Assays showed an increase in viability and osteogenic differentiation of ASCs in comparison with the hydrogel only and similar results in comparison with BMP loaded hydrogel, both *in vivo* and *in vitro*. Moreover, all the studied hydrogels had a positive effect on bone healing in the defect sites and were biologically degradable by collagenase.

Graphene, a two dimensional carbon structure that presents unique physical properties such as high surface area, high mechanical strength, high electrical conductivity and ease of functionalization, has been recently reported to be able to form hydrogels and to promote *in vitro* osteogenic differentiation and proliferation of stem cells [136,137,138]. Lyu *et al.* examined the osteoinductivity of hASCs loaded self-supporting graphene hydrogels (SGH) obtained by electrostatic stabilisation of graphene and its gelation by filtration [139]. They found that in comparison to conventional graphene and carbon fibre films, SGH films had higher mechanical strength, flexibility, cell viability (after one day culture, 95.43% ± 0.96%), mineralisation and osteoinductivity. This finding opens new avenues in the development of hydrogels for bone regeneration applications.

#### 3.3.3. Stem Cells from Human Exfoliated Deciduous Teeth

Stem cells from human exfoliated deciduous teeth (SHEDs) have been recently identified as an alternative source of multipotent adult stem cells because of their capacity to differentiate into various cell lineages: neural cells, odontogenic cells, osteogenic cells and adipocytes [140,141]. Moreover, they were reported to have a higher proliferation rate compared to MSCs and dental pulp stem cells and to be easier and more convenient to isolate [140,141,142]. Unfortunately, few studies about the loading of SHEDs into hydrogels have been published until now, underling the importance of further research in this field.

Su *et al.* [140] investigated the effects of chitosan/gelatin/β-glycerophosphate thermosensitive hydrogels containing strontium phosphate or tricalcium phosphate (TCP) on the osteogenic differentiation of SHEDs *in vitro*. Strontium has been added to the scaffold since it has been shown to positively influence bone formation and inhibiting bone resorption [143,144]. Scanning electron microscopy analysis of the surface revealed that all the studied hydrogels presented homogenous porous structures (100–300 µm) with high interconnected channels that had high cytocompatibility. Nevertheless, the presence of bioceramic supplements, allowed continuous proliferation of cells for longer cultivation times (14 days). Strontium hydrogels also significantly increased calcification and expression of osteogenic genes compared to tricalcium phosphate.

#### 3.3.4. Embryonic Stem Cells

Embryonic stem cells (ESC) are pluripotent stem cells derived from the inner cell mass of blastocysts and they can differentiate to form cell populations derived from ectoderm, mesoderm and endoderm, hence, any cell type [145]. They possess a nearly unlimited self-renewal capacity and their growth is not restricted by contact inhibition and proliferative senescence. As reported by Li *et al.*, ESCs are a promising cell source for regenerative medicine, even though some challenges need to be overcome, such as achieving large-scale expansion culture systems, mainly due to the involvement of animal components [146,147]. Although the propagation of embryonic stem cells has usually been carried out in two-dimensional (2D) systems, 3D scaffolds would facilitate convenience in transplantation and consistency in cell performance and scalability in number [148]. Hence, the use of natural and synthetic hydrogels for hESC propagation in 3D has been proposed [148,149,150,151,152]. However, the study of ESCs properties and propagations on hydrogels for bone tissue engineering needs to be further explored.

Zur Nieden *et al.* studied the chemical modification of gelatin hydrogels through glyceraldehyde cross-linking to provide a suitable scaffold for ESC osteogenesis [115]. On one hand, gelatin was used as the main component of the matrix for its desirable properties: low toxicity, easy sterilisation and limited higher ordered protein structure. On the other hand, glyceraldehyde is an ideal cross-linker since it is non-toxic and makes gelatin stable at 37 °C. The obtained gel was able to provide an initial soft non-adhesive surface for promoting the formation of embryoid bodies and to provide a harder surface that gradually disappeared as osteoblasts differentiated.

## 4. Concluding Considerations

The wealth of research into hydrogels formulations for bone regeneration applications demonstrates the great potential of these systems. Hydrogels fulfill many of the requirements of ideal scaffold such as injectability, biocompatibility and biodegradation. Furthermore, they can easily be chemically modified or co-formulated with other components that bestow further properties such as osteoconduction, osteoinduction and oesteogenicity. Hydrogels can be composed of a great variety of synthetic and natural polymers and their capacity to function as a diffusion barrier for both small and large molecular weight drugs has been widely exploited for controlled drug delivery applications. All proposed systems seem to provide incremental improvements to the performance of hydrogels, but, perhaps, the development of fundamentally innovative ideas is the key to moving to clinically applicable formulations. Approaches such as bioprinting or the development of fundamentally new hydrogels such as those formed by graphene oxide could open up novel and more promising avenues towards the successful clinical use of composite hydrogels for bone regeneration.

## Figures and Tables

**Table 1:** Mechanical properties of composite hydrogels containing inorganic components.

Scaffold Composite Components Organic Inorganic		Ratio	Compressive Strength (MPa)	Compression Modulus (KPa)	Elastic Modulus (MPa)	Reference
Poly(acrylamide)	nHA	85:15	35.8	-	-	[52]
Silk fibroin	nHA	85:15	-	109.8	-	[55]
Agarose	nHA	65:35	390	-	1104.4	[57]
Oxidized alginate-gelatin-BCP	Spherical HA	65:35	-	2.45 dry 0.05 wet	-	[58]
Chitin	nHA	75:25	0.3	-	0.3	[60]
Gellam gum	Bioglass	50:50	-	-	1.2	[71]
PEG	Bioglass	80:20	2.5	-	8	[72]
Chitosan/silica	f-CNTs	98:2	-	-	552	[77]
PPF	Nano Carbon	0.2	-	2061	-	[80]
Cortical bone		-	130–180	-	12,000–18,000	[82]
Trabecular bone		-	4–12	-	100–500	

**Table 2:** Growth factors employed in bone tissue engineering applications [36].

Growth Factor	Mechanism of Action	Limitations
**BMP-2** (Bone morphogenic protein)	Induces osteoblasts proliferation and mesenchymal cells (MSCs) differentiation Induces VEGF-A secretion therefore has a role in angiogenesis ^1^	Needs to be delivered in a controlled manner Variable outcomes have been seen in humans Limited capacity to initiate vascular proliferation
**VEGF** (Vascular endothelial growth factor)	Induces endothelial cells mitogenesis Attracts MSCs and induces their differentiation	Delivered alone they lead to the inability to produce organized bone regeneration
**PDGF** (Platelet derived growth factor)	Attracts cells that stabilise growing vasculature Recruits MSCs Upregulates VEGF production	
**FGF** (Fibrobast growth factor)	Involved in the formation of new capillaries	
**IGF** (insulin like growth factor)	Involved in adult neo angiogenesis	

^1^ pro- or anti- osteogenic effect can be observed depending on the type of BMP used and the type of cells targeted.

## Abbreviations

The following abbreviations are used in this manuscript:

ASCs	adipose-derived stem cells
BFGF	basic fibroblast growth factor
BMP	bone morphogenic protein
BPs	bisphosphonates
BTE	bone tissue engineering
C60	fullerenes
CaP	calcium phosphate
CDA	calcium deficient apatite
CNTs	carbon nanotubes
Cs	chitosan
ECM	extracellular matrix
ESC	embryonic stem cells
f-CNT	functionalized-carbon nanotubes
FGF	fibroblast growth factors
GG	gellan gum
GNPs	gold nanoparticle
HA	hyaluronic acid
Hap	hydroxyapatite
HMG-CoA	3-hydroxy-2-methylglutaryl-CoA
hMSCs	human mesenchymal stem cells
HSC	hematopoietic stem cells
IGF	insulin like growth factor
MSC	mesenchymal stem cells
MSNPs	molybdenum di-sulfite nanoplatelets
MWNTs	multi-wall nanotubes
nHap	nanohydroxyapatite
PDGF	platelet-derived growth factors
PEG	polyethylene glycol
PLGA	poly(lactide-*co*-glycolide)
PPF	poly(propylene fumarate)
PTH	parathyroid hormone
SGH	self-supporting graphene hydrogels
SHEDs	stem cells from human exfoliated deciduous teeth
SIM	simvastatin
SWNTs	single-wall nanotubes
TCP	tricalcium phosphate
TE	tissue engineering
TGF-β	transforming growth factor
TNF-α	tumour necrosis factor-α
US-tubes	ultra-short single-wall nanotubes
VEGF	vascular endothelial growth factor
Zol	zoledronate
